# Drugs Many: Remedies Few

**Published:** 1895-08-10

**Authors:** 


### The Hospital

An Institutional Journal of

The Medical Sciences and Hospital Administration, with

AN EXTRA NURSING SUPPLEMENT.

Vol. XVIII.—No. 463.] [Aug. 10, 1895.

### Drugs Many: Remedies Few.

"New drugs are added every day for the benefit chiefly of those who do not know how to employ the old ones." Such is the verdict of Sir William Broadbent, pronounced in the presence of his professional brethren assembled in annual congress under the auspices of the British Medical Association. By way of set-off to this may be placed the opinion of the average patient, who plumes himself upon his family physician or favourite consultant because the learned gentleman is so thoroughly "up-to-date," and so prompt in the application of "all the new things." It will probably be granted that the judgment of Sir William Broadbent on this lively question of "new or true" is weightier than that of the average patient, and if the present were an age of respect for authority, the question might be considered settled in that sense. But authority being one of the things that are out of date, and all men, inexperienced medical men in especial, being much enamoured of the so-called "new" because it is new, it is well worth while to give a little serious study to the subject.

At the outset it is desirable to remind ourselves that *Timeo Danaos et dona ferentes* has its application in medicine as in other departments of human activity. It is necessary to consider the source and origin of most of the "new remedies" which are attempted to be introduced into practice. For the most part they are the products of enterprising manufacturers. Now there can be no reason why the manipulators of chemicals should not use the utmost possible energy and enterprise in the pursuit of their calling. In so far as they devote labour and money to the bringing under investigation of all kinds of substances belonging to the animal, vegetable, or mineral worlds, they do well, and only well. So doing they fulfil, not only a useful, but an indispensable function. Even when they go farther, and so far trench upon the work of the physician as to make combinations of drugs of known action, with the view of evolving a combined product which shall be of definite therapeutic service, they are still not merely to be excused, but even commended. The scientific and enterprising manufacturing chemist is a development for which medicine must be grateful.

But there is another side to the question, and it is that other side which Sir William Broadbent insists upon thrusting before our eyes when he tells us, with a sternness not unmixed with humour, that "new drugs are added every day for the benefit chiefly of those who do not know how to employ the old ones." What, then, in a word, is the point of Sir William Broadbent's judgment? It is this, that many men never set themselves to prove experimentally for themselves the value of any drug or drugs, and so they never come to a condition of mind in which they employ remedies with confidence, precision, and success. The story of King David's sling and stone has a priceless application in medicine. It is infinitely better for a man to confine his practice to the use of a dozen drugs of which he has long and intelligently studied the various applications, than to employ a thousand of most of which his knowledge is merely guess work. In so affirming we are using no recondite philosophy, but only the most obvious common sense. In other words, we insist that a man shall study the results of the drugs he uses; shall study them philosophically, by careful observation and comparison, and by judicial decision arrived at by the operation of his own mind. It is the ruin of modern medicine that men do not use their minds and base their work on the immovable foundation of their own proved convictions. They are not even so steady as kites in the air, which are at least moored to the earth by strings. Their counterpart is the thistle-down; and they are carried here, there, and everywhere, nobody knows where, by every therapeutic puff of wind that blows.

Is there, then, no place for new remedies and new methods in modern medical practice? There is room of the amplest; room and to spare. What we insist upon is this twofold principle, that the old which is tried and proved shall be loyally preserved, that well-known drugs shall be retained, and that the new shall always, in every case, and by every individual, be subjected to continuous and competent examination and proving. This, we say, is not recondite philosophy; it is the most elementary common sense. But it is for the lack of this elementary common sense in daily practice that thousands of practitioners are soldiers bearing rifles but who cannot shoot, sailors in command who cannot navigate their own ships. The third-rate practitioner, who has not gained a just self-confidence, by reason of thoroughness and success in practice, always hankers after the reputation of being thoroughly "up-to-date." The British Medical Association will not have met in London in vain if all men of this order, and all their patients also, will seriously lay to heart Sir William Broadbent's words, that "new drugs are added every day for the benefit chiefly of those who do not know how to employ the old ones."

